# Changes in patients characteristics and service provision in liaison psychiatry during the COVID-19 pandemic

**DOI:** 10.1192/bjo.2021.895

**Published:** 2021-06-18

**Authors:** Frederica Passos, Miguel Constante, André Delgado, Maria João Heitor

**Affiliations:** 1 Departamento de Psiquiatria e Saúde Mental, Hospital Beatriz Ângelo; 2 Departamento de Psiquiatria e Saúde Mental, Hospital Beatriz Ângelo, Instituto de Saúde Ambiental, Faculdade de Medicina, Universidade de Lisboa

## Abstract

**Aims:**

The SARS-CoV-2 pandemic has led to core changes in the healthcare systems worldwide in terms of access, resources and patient's management. Patients admitted to a general hospital with COVID-19 are at a higher risk for developing or exacerbating various psychiatric disorders, due to the virus brain tropism and associated psychosocial factors. However, there are still limited data regarding the psychiatric needs and mental health care of these patients. The present study seeks to evaluate the changes in liaison psychiatry, patients’ characteristics and outcomes, brought on by SARS-CoV-2.

**Method:**

We retrospectively analyzed liaison psychiatry patients’ characteristics during the second wave of COVID-19 pandemic, between October and December 2020, in comparison with the same trimester pre-COVID-19 in 2019.

**Result:**

At admission to hospital, we found no differences between liaison psychiatry pre-COVID-19 (n = 96) and COVID-19 period (n = 114) groups, for age (p = 0.35), gender (p = 0.96), or hospital resource utilization in the previous year : number of hospital admissions (p = .40), Accident and Emergency presentations (p = 0.61), outpatients clinics (p = 0.20). There was, however, a significant association between pre-COVID-19 vs COVID-19 period groups and psychiatric diagnosis during hospitalization (Χ2(5) = 11.56, p = 0.04), influencing overall respective rates of delirium/dementias (29.2 vs 42.1 %), mood disorders (18.8 vs 26.3 %), anxiety and stress related disorders (22.9 vs 14.0 %), psychoactive substance use disorders (12.5 Vs 9.6 %) and psychotic disorders (10.4 vs 2.6 %). Mortality rate in pre-COVID-19 Vs COVID-19 period increased from 5.2 % to 15.8 % (Χ2(1) = 5.98, p = 0.01), mostly due to increased mortality in COVID-19 patients (7.3 vs 30.3 %, p < 0.001). Referrals to liaison psychiatry increased by 18.8 % during the COVID-19 period, 28.8 % of referred patients were positive for SARS-CoV-2 and time to referral was positively correlated with hospital length of stay (ρ = 0.60, p < 0.001). In the COVID-19 period, fewer patients were referred to outpatient psychiatric follow-up after discharge from hospital (p < 0.001), and delayed discharges for social reasons were of shorter duration (p = 0.001).

**Conclusion:**

Our findings are consistent with emerging data on comorbid or superimposed mental illness in hospitalized patients with COVID-19, showing a strong impact in neuropsychiatric mani-festations, clinical prognosis and mortality, as well as clinical management. Consideration should also be given to adequate staffing of psychiatry liaison services during the pandemic, in order to attend and safely manage general hospital inpatients mental health needs.

